# On the Operation of Cupping

**Published:** 1809-09

**Authors:** 


### *To the Editors of the Medical and Physical Journal.*

GENTLEMEN,

NOT doubting that the desire of information on any subject of utility in the practice of medicine, will be a sufficient apology for wishing to occupy a small portion of your valuable pages, I proceed to request of some of your numerous Readers, who may be so fortunate as to have acquired dexterity in the art of taking blood by cupping, to detail that operation with minuteness, and to explain on what circumstances the success of it depends. In London, this operation is consigned to particular persons, some of whom have probably, from frequent practice, acquired superior excellence in performing it; but though the operation appears so easy, I have been disappointed that after much expence on the subject, and considerable pains taken, I seldom succeed in procuring any considerable quantity of blood. I should therefore be happy to be instructed by some of your liberal and better informed Correspondents, from what particulars my failure may proceed, and what precautions would be likely to ensure success.

In a late case of pleuritis, wherein general bleeding had been carried as far as appeared to be safe; on account of frequent stitches in the side still occuring, the scarificator was applied four or five times, and though by the adhesion of the glasses, the vacuum appeared to have been pretty complete, not more than an ounce or two of blood was procured, and notwithstanding the case ended favourably, I felt mortified that so little blood could be obtained.

I have

I have used scarificators made by eminent London instrument-makers; and in the above case the lancets had not been used since being newly ground, notwithstanding which they did not appear to cut well, and this defect seemed to depend on a want of sufficient strength in the spring, which although it appeared pretty strong in cocking the instrument, was yet too weak to carry the points of the lancets clean through the integuments, while the instrument was pressed upon, so that the brass box which contains the lancets, was elevated by them as by levers, instead of keeping its situation; or if sufficient pressure was applied to prevent that circumstance, the lancets were arrested in their progress, and obliged to be pulled round by the trigger, making the operation clumsy, painful, and tedious; and this fault of the instrument is apparently the chief impediment to success; the best way of making the vacuum appears to be by the flame of a spirit lamp. Is the form and size of the glass of particular importance? That used in the present instance, is at the mouth 1 inch $\frac{7}{8}$ wide, its depth is 2 inches $\frac{7}{8}$, and about an inch above the mouth it bulges out, and terminates at the depth described in a globular form.

Country practitioners may often have occasion to perform this operation, and I have too good an opinion of it to abandon it entirely as some have done, partly from esteeming it of little importance, but more from their own unskiiful-ness in performing it. I have known it of considerable utility, even when a small quantity of blood was obtained, which it is probable may arise from a considerable quantity of blood having been brought to the surface, and thus relieving the deep-seated membranous part, on which principle dry-cupping was formerly practised.

I am, &c.

A Country Practitioner.

P. S. As it is always of importance to confirm the succesful practice of an easy method of cure in a troublesome disease, I beg leave to mention, that I have lately succeeded in the cure of three cases of hydrocele of the tunica vaginalis testis, by the injection of a mixture of equal parts of Port wine and water; in the last case, the inflammation, instead of producing immediate adhesion of the sides of the cavity, ended in a suppuration, apparently between the dartos and tunica vaginalis, which latter substance appeared to come away in tough ragged sloughs; and the case occupied from three to four weeks in the cure, which was in the end complete.

I wish

I wish to observe, that it greatly facilitates this operation to make a short incision through the integuments with the shoulder of a lancet, so as to lay bare the tunica vaginalis, through which the trocar passes with much greater ease than when it is pushed through the integuments which it is apt to drive before it, and thus gives more pain to the patient, and trouble to the operator.

---

### Remarks by the Editors.

There are two or three hints we would readily offer to our "Country Practitioner." We shall be glad if any other correspondent will enlarge on the subject, and if he thinks it necessary, correct our remarks, which cannot strictly be called practical, as we have had few opportunities of performing the operation.

It is of the utmost consequence that the glasses be perfectly dry as well as warm, when applied. This is particularly necessary when the glasses are applied after the scarification is made.

It cannot be necessary to add, that the rarification by the heat should be accomplished immediately over the spot to which the glass is to be applied.

The operator should have glasses of different sizes, and even of different forms, to suit the part to which he applies them.

As often as a glass is removed for the application of a fresh one, the orifices should be carefully washed with warm water and a sponge, that no coagulum may remain on them.

All these remarks are obvious enough, but there is one more which does not occur so often as we might expect.

If the operation is performed on fat subjects, and particularly on the surface most loaded with fat, the process will be tedious in spite of all our caution. This more commonly occurs in the parietes of the abdomen; but the whole front surface of the trunk, particularly in females who are not young, is often so charged with obesity, as to be scantily provided with blood vessels.

